# 3D Porous Graphene Based Aerogel for Electromagnetic Applications

**DOI:** 10.1038/s41598-019-52230-5

**Published:** 2019-10-31

**Authors:** Hossein Cheraghi Bidsorkhi, Alessandro Giuseppe D’Aloia, Alessio Tamburrano, Giovanni De Bellis, Andrea Delfini, Paolo Ballirano, Maria Sabrina Sarto

**Affiliations:** 1 Department of Astronautical, Electrical and Energy Engineering, Sapienza University of Rome, via Eudossiana 18, 00184 Rome, Italy; 2 Research Center on Nanotechnology Applied to Engineering of Sapienza (CNIS), Sapienza University of Rome, P. le Aldo Moro 5, 00185 Rome, Italy; 3 Department of Earth Sciences, Sapienza University of Rome, P. le Aldo Moro 5, 00185 Rome, Italy

**Keywords:** Graphene, Graphene, Magnetic properties and materials, Aerospace engineering, Electrical and electronic engineering

## Abstract

Lightweight multifunctional electromagnetic (EM) absorbing materials with outstanding thermal properties, chemical resistance and mechanical stability are crucial for space, aerospace and electronic devices and packaging. Therefore, 3D porous graphene aerogels are attracting ever growing interest. In this paper we present a cost effective lightweight 3D porous graphene-based aerogel for EM wave absorption, constituted by a poly vinylidene fluoride (PVDF) polymer matrix filled with graphene nanoplatelets (GNPs) and we show that the thermal, electrical, mechanical properties of the aerogel can be tuned through the proper selection of the processing temperature, controlled either at 65 °C or 85 °C. The produced GNP-filled aerogels are characterized by exceptional EM properties, allowing the production of absorbers with 9.2 GHz and 6.4 GHz qualified bandwidths with reflection coefficients below −10 dB and −20 dB, respectively. Moreover, such aerogels show exceptional thermal conductivities without any appreciable volume change after temperature variations. Finally, depending on the process parameters, it is shown the possibility to obtain water repellent aerogel composites, thus preventing their EM and thermal properties from being affected by environmental humidity and allowing the realization of EM absorber with a stable response.

## Introduction

Over the past few years, the tremendous development of communication technologies and electronic devices based on densely packed systems, has fueled the research on electromagnetic (EM) wave absorption^1^. In fact, EM interference issues increased exponentially and nowadays EM pollution is a serious problem that could compromise equipment performances and electronic reliability, leading to lifetime degradation, loss of energy and data, in signals and storage devices^2^. For instance, undesired EM radiation can be detrimental to aeronautics safety by interfering with on board electronic control systems and affecting the signal quality in high-speed communication devices^3^. Therefore, EM wave absorption is crucial and large efforts have been made in the development of new high-performance absorbing materials. As an example, the use of such materials in emerging satellite communication systems improves receiver’s signal quality, through noise suppression^3^. However, traditional absorbing materials, such as ferrite^4^, magnetic metal powders^5^ and ceramics^6^, have a limited use in emerging space and aerospace industry, as well as in electronic and communication technologies, due to their high density, heavy weight and environmental degradation^7^. In fact, weight reduction is mandatory in space and aerospace industry, and good chemical, physical and thermal resistance are key requirements in forthcoming flexible and miniaturized electronic devices^8^.

To date, varieties of carbon-based conductive polymer composites have been widely investigated in order to replace conventional absorbing materials, since they combine good mechanical and electrical properties with typical polymeric characteristics, such as lightweight, high formability and resistance against corrosion^8^. Indeed, carbon nanotubes, carbon nanofibers and graphene nanoplatelets (GNPs) have been considered as conductive fillers in polymer nanocomposites for their remarkable structural, mechanical, and electrical properties. Among them, GNPs gained most attention as nanofillers in thermosetting polymer composites, since they are extremely low-cost and easily processable^8^. For instance, a broadband polymeric absorber consisting of an epoxy-based vinyl ester resin filled with GNPs has been proposed in^9^. GNP/epoxy composites were developed and characterized in terms of electrical and electromagnetic properties in order to assess potential applications of these materials as conductive coatings and microwave absorbers for frequencies up to 18 GHz^10^.

Even though large efforts have been made, the development of lightweight multifunctional EM absorbing materials for space and aerospace applications is still a challenge. Actually, it is hard to reach a satisfactory compromise between thermal, mechanical and EM properties in GNP-loaded epoxy composites^11^.

Nevertheless, impressive advances have been made in porous and aerogel structures, leading to a substantial reduction of the composite density and weight^12^. Carbon aerogels, consisting of sponge-like interconnected networks of porous carbon, grabbed even more attention due to their lightweight along with excellent electrical and thermal properties^12–15^. Indeed, several authors investigated the production and the properties of carbon aerogels for EM applications. Such materials are mainly divided into two groups: graphene-based aerogels^12,15–17^ and graphene based polymer composite aerogels^18–20^. The first ones are generally characterized by excellent EM properties but low mechanical properties, flexibility, ductility and thermal stability^15^. Moreover, their production process often requires the use of dangerous chemicals, such as acids or bases, and, additionally, can be quite expensive.

Despite being generally cheaper, graphene-based polymer composite aerogels, feature higher mechanical properties, flexibility and easier processability than graphene based aerogels^18–20^. Among them, the ones using polyvinylidene fluoride (PVDF) as polymer matrix are showing a growing interest due to the exceptional chemical resistance and thermal, mechanical and physical properties of PVDF^21,22^. Furthermore, the hydrophobic characteristics of PVDF are of particular relevance for the realization of cost-effective self-cleaning, anti-biofouling and humidity resistant surfaces^23,24^.

H. Wang and co-workers presented a novel technique to manufacture porous nanocomposites based on PVDF and multiwall carbon nanotubes (MWCNTs) for EM shielding purposes in the frequency range 8–18 GHz^18^. A PVDF polymeric matrix was used also by X. Ma *et al*. for their porous hydrophobic polymer composite with enhanced EM shielding and self-cleaning capabilities^19^. An aqueous based approach for fabrication of PVDF porous composites filled with MWCNTs, with improved EM properties, has been reported in^20^. However, it should be pointed out that the composites investigated in^18–20^ are loaded with a relatively high percentage of nanofiller, and that the EM absorption properties are out of the scope of these studies.

Even though GNPs are extensively used as fillers in graphene-based composites^8–11^, to date very few works deal with porous PVDF-GNP composite aerogels^25–27^. B. Zhao *et al*. found a method to fabricate PVDF-GNP nanocomposite foams for shielding applications^25^. In^26^ a PVDF matrix is loaded with graphene and silver nanoparticles, while in^27^ a novel polyacrylonitrile/PVDF-co-hexafluoropropylene/GNP nanocomposite foam is presented. All these composites show improved EM properties, but the EM wave absorption capabilities are not investigated, neither the mechanical and thermal ones. Moreover, chemical or physical filler functionalizations are needed and cost-effective production processes are required^25–27^, involving the use of dangerous chemicals or critical raw materials, such as silver nanoparticles^26^.

The aim of this paper is to overcome these bottlenecks, producing a cost-effective lightweight 3D porous graphene-based aerogel for EM wave absorption, characterized by high porosity, good mechanical properties, excellent thermal and electrical conductivities. The produced graphene-based aerogels consist of a PVDF polymer matrix filled with GNPs, without any chemical modification or functionalization, either of GNPs or of the polymer chains. Thus, the production process is scalable, relatively simple, and does not involve the use of any acids, bases or surfactant.

The produced aerogels are characterized by remarkable chemical and physical resistances along with thermal stability and mechanical, thermal, electrical and EM properties. In particular, they allow the production of extremely high-performing broadband radar absorbing screens. In fact, with a thickness of only 2.2 mm, it is possible to obtain an absorbing panel with a reflection coefficient’s bandwidth lower than −10 dB of about 9 GHz centered at ~13.5 GHz, and of more than 6 GHz for reflection coefficient lower than −20 dB. This EM absorption performance is comparable with the one resulting from graphene foam^28^ and particularly relevant with respect to the ones obtained with graphene-silicon carbide aerogels^29^ and with other porous polymer nanostructures^30–32^. In fact, Y. Jiang *et al*.^29^ claimed a reflection coefficient bandwidth at −10 dB limited to 4.7 GHz in the 8 ÷ 18 GHz frequency range. A. Xie *et al*.^30^ presented a helical conducting polypyrrole nanostructure characterized by a 7.04 GHz qualified bandwidth with reflection coefficient below −10 dB. L. Wang and coworkers produced hierarchically porous cobalt based zeolitic imidazolate frameworks with the aim of obtaining honeycomb porous composites for radar absorption having a −10 dB reflection coefficient bandwidth limited to 5 GHz^31^. In^32^ a coaxial silver-polypyrrole porous nanocomposite having a qualified bandwidth of 5.88 GHz is presented.

In addition to the wideband EM properties, the samples produced in this study show exceptional thermal conductivities and stability, i.e. a negligible volume change after temperature variations. All these characteristics make the proposed aerogels good candidates for EM absorption in aerospace, space and electronic applications. Moreover, depending on the process parameters, the PVDF/GNP aerogels can be rendered water repellent. This quality is particularly relevant for electronic packaging, since the EM and thermal performances of these materials will not be affected by environmental humidity.

In brief, the novelty of this work is fivefold. First, the produced GNP-filled composite aerogels are characterized by exceptional EM properties, allowing the production of absorbing panels with a 9.2 GHz and a 6.4 GHz qualified bandwidths with reflection coefficients below −10 dB and −20 dB, respectively, in the 8 ÷ 18 GHz frequency range. Second, the obtained aerogels show exceptional thermal conductivities without any visible volume change after temperature test. Third, depending on the process parameters, it is possible to obtain water repellent aerogel composites, thus avoiding their EM and thermal properties being affected by environmental humidity. Fourth, the new production process proposed is simple and scalable, it does not involve use of any acids, bases or surfactant and it does not require a filler functionalization, neither a chemical one, nor a physical one. Fifth, we show that the proper setting of the processing temperature while mixing PVDF-DMF solution and PVDF enable to tune the thermal, electrical, mechanical properties of the aerogel, since it affects the incorporation of GNPs into the polymer matrix. All of these aspects enable to overcome the limitations of state of art EM wave absorbers for aeronautical, space and electronic packaging applications.

## Results

**Figure 1:**
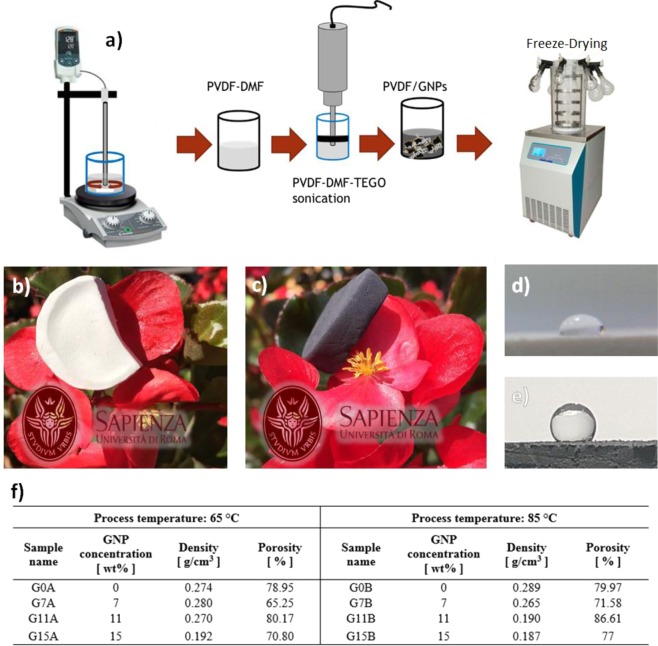
(**a**) Schematic procedure for the production of PVDF-GNP nanocomposite aerogel samples. Lightweight aerogel samples made of neat PVDF (**b**) and GNP-loaded PVDF (**c**) over flower petals. Water drop over the hydrophilic of (**d**) or hydrophobic (**e**) surfaces of aerogel samples, made of PVDF or GNP-loaded PVDF, respectively. (**f**) List of the produced graphene-based aerogel samples with their GNP concentrations, density and porosity values.

Following the procedure summarized in Fig. 1a), eight different PVDF-GNP nanocomposite aerogel samples were fabricated. They are characterized by a GNP content varying between zero and 15% wt. with respect to the PVDF amount and by two different process temperatures, namely 65 °C and 85 °C. Pictures of the produced aerogel samples made of neat PVDF and GNP loaded PVDF are shown in (Fig. 1b,c), respectively: they are extremely lightweight and homogeneous. Furthermore, through the proper setting of the process parameter described in the Method section, the produced samples can be rendered either water-repellent or water-absorbent, as shown in (Fig. 1d,e), respectively.

All the produced samples are listed in (Fig. 1f) and named as follows: the letter G stands for graphene-aerogel, followed by a number indicating the GNP weight concentration (0%, 7%, 11% or 15% wt.), while the last letter is representative of the process temperature, i.e. 65 °C (letter A) or 85 °C (letter B). The density and the average porosity of the considered aerogel samples are reported in (Fig. 1f). It can be noticed that the density of the produced samples is strongly affected by the GNP content. The porosity of neat PVDF samples, G0A and G0B, is around 79%. It reaches its minimum with the addition of 7% wt. GNP and its maximum value with the addition of 11% wt. GNP, regardless of the process temperature.

**Figure 2:**
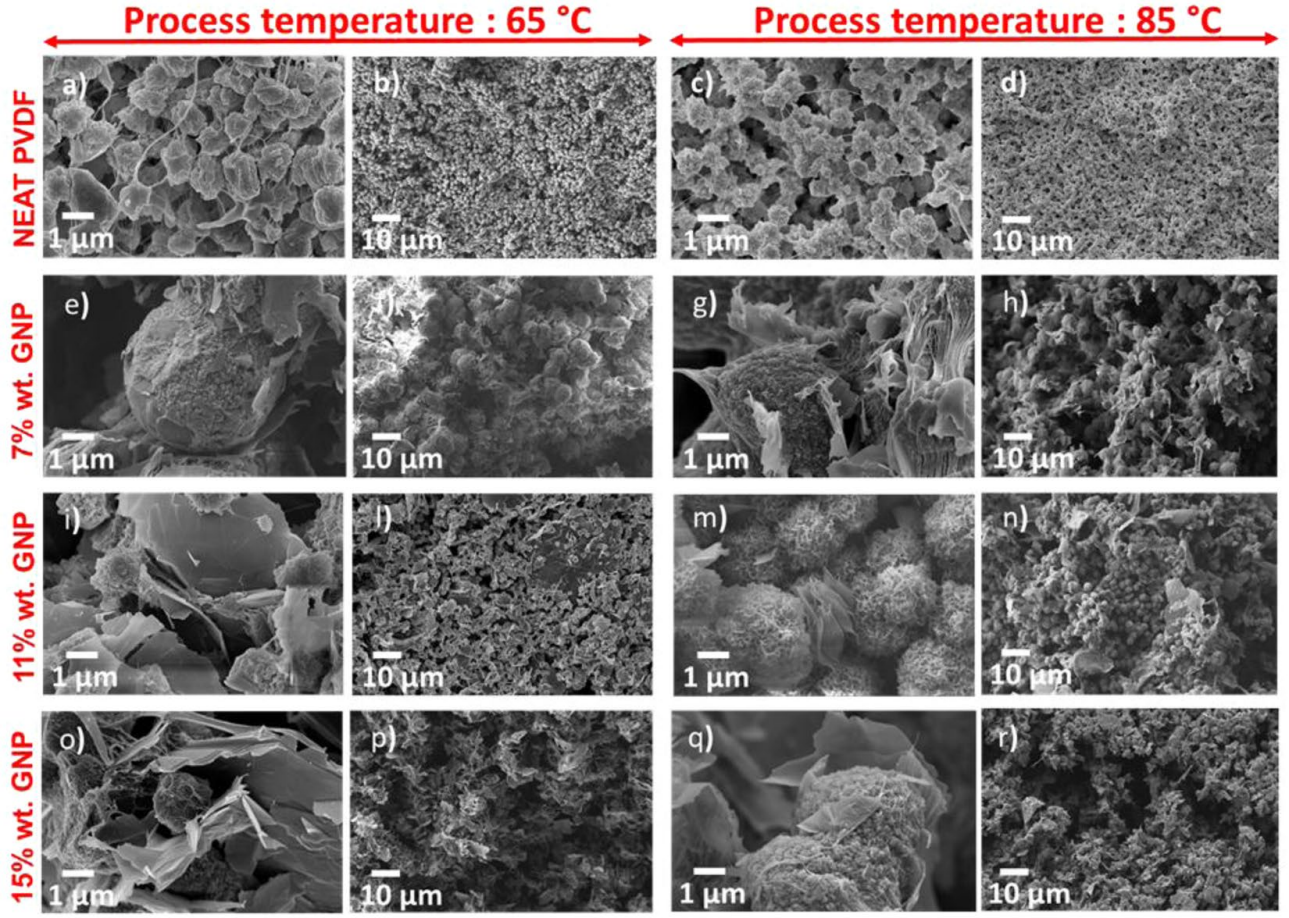
SEM images at different magnifications of the produced aerogel samples with different content of GNPs, as reported in Fig. 1(f). (**a**,**b**) G0A; (**c**,**d**) G0B; (**e**,**f**) G7A; (**g**,**h**) G7B; (**i**,**l**) G11A; (**m**,**n**) G11B; (**o**,**p**) G15A; (**q**,**r**) G15B.

Figure 2 shows SEM images of all the produced composite aerogels acquired at different magnifications.

The neat PVDF aerogel composite samples, G0A and G0B, show a porous structure with spherulites having average diameter around 1.5 µm and connected each other by PVDF nano-fibers. Moreover, a homogenous pore distribution, with pore size around 1 µm or below, is noticed.

The GNP loaded PVDF aerogel samples are characterized by a good integration of GNPs inside the polymer matrix. The GNP surfaces are uniformly dispersed into PVDF and GNPs clearly coalesce into the PVDF, giving rise to a new material structure.

From the comparison of SEM images of A-type and B-type samples, we observe that GNPs are incorporated into PVDF when the process temperature is set at 85 °C (i.e. B-type samples): it results that B-type aerogels are characterized by a 3D-framework in which the two different phases (i.e. the polymer and graphene) are fully integrated and act more as a unique porous structure. Differently, in the A-type aerogel, we notice that GNPs are well distributed among PVDF spherulites; then, it results that these samples for a lower GNP content (i.e. 7% wt.) are characterized by a 3D-polymer framework surrounded by GNPs, whereas for a higher GNP content (i.e. 15% wt) they are formed by a 3D-GNP skeleton embedded in the polymeric matrix.

Such observations are in line with the fact that in B-type samples, the average size of the spheroids is nearly the same for all the considered GNP weight concentrations. On the other hand, in A-type samples the morphology is more affected by the GNP content than in B-type samples. In particular, sample G7A has spheroidal particles with an average diameter of about 5 µm, much greater than that observed in the other samples, in which spheroids are about 1.5 µm in diameter. Moreover, it can be noticed that in sample G7A spherulites are poorly interconnected through a few PVDF filaments, which are not electrically conducting. On the other hand, in all the other samples loaded with a higher amount of GNPs, spherulites are well interconnected through the graphene flakes, which are electrically conducting.

**Figure 3:**
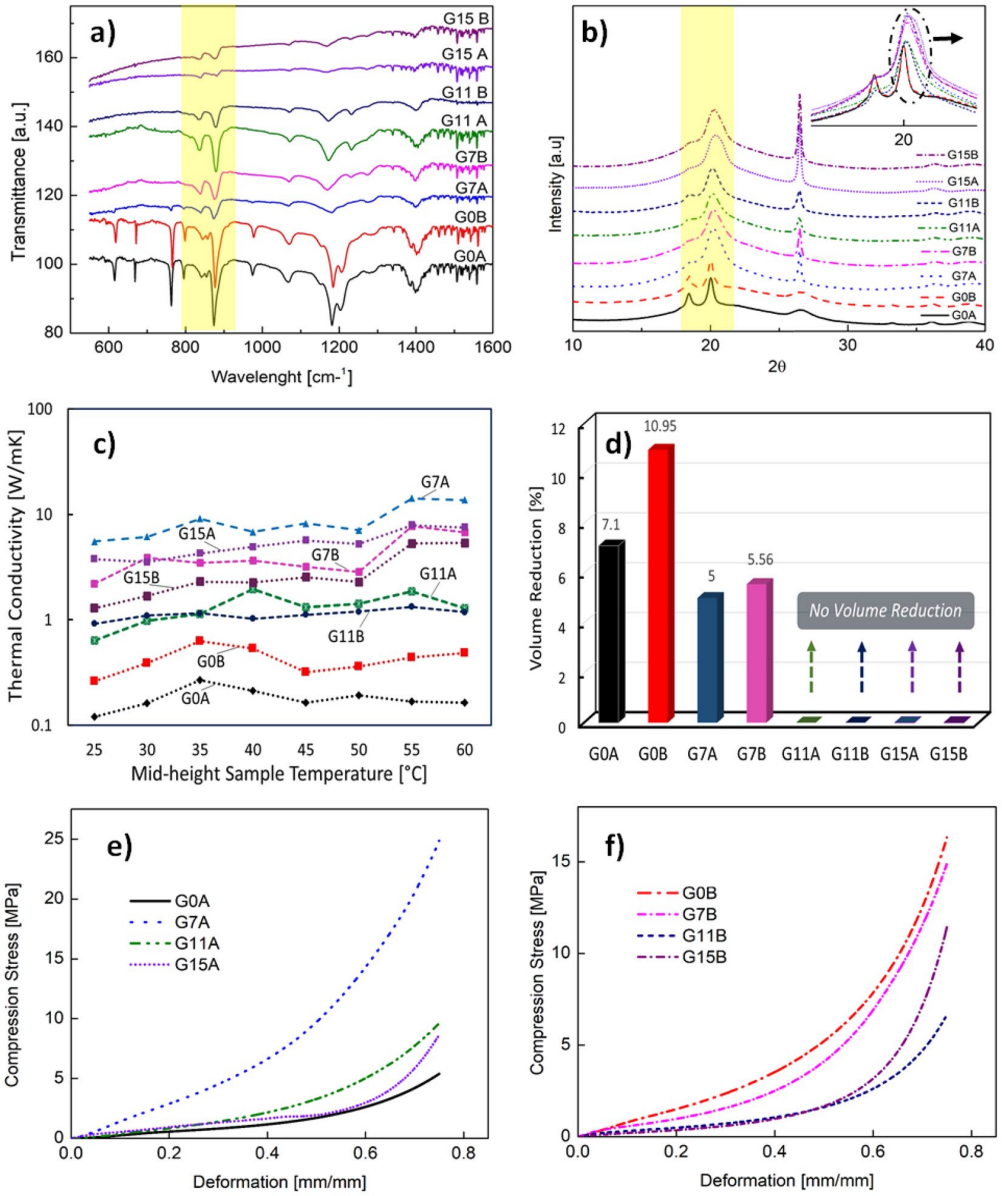
Results of characterization tests performed on the produced aerogel samples loaded with different GNP content, as reported in Fig. 1(f). (**a**) FTIR; (**b**) XRD; (**c**) Thermal conductivity vs. mid-height sample temperature. (**d**) Percent volume reduction after thermal conductivity test; (**e**,**f**) compression test vs. deformation. The inset of (**b**) highlights the peak shift due to β phase transition.

Figure 3a) shows the Fourier transform infrared (FTIR) spectroscopy results obtained from neat PVDF and GNP-loaded PVDF aerogel samples. According to the outcome of FTIR analysis of neat PVDF samples, wave bands, recognized at 484 cm^−1^ (wagging), 531 cm^−1^ (bending), 614 cm^−1^ (skeletal bending), 762 cm^−1^ (bending), 794 cm^−1^, 973 cm^−1^ (twisting), 1183 cm^−1^ (stretching) and 1203 cm^−1^ are assigned to the PVDF *α*-phase, as reported in^33,34^. By adding GNPs, the 973 cm^−1^ wave band almost disappears, the intensity of the previous peaks diminishes noticeably, and absorbance bands with mighty intensity at 509 cm^−1^ and 840 cm^−1^ appear. Thus, the last absorbance bands are related to the PVDF *β*-phase^35^.

The crystal structure of neat and GNP-loaded PVDF samples was investigated through X-ray powder diffraction (XRPD) technique, and the results are reported in (Fig. 3b). As stated in recent studies^36–39^, the reflection peaks of PVDF are at 2*θ* = 17.76°, 18.42°, 20.00°, 26.68°, 33.14°, 35.96° and 38.7°, which are allocated at (100), (020), (110), (021), (130), (210), and (002) planes, respectively. These characteristic peaks belong to PVDF *α*-phase, and the main crystalline peak of PVDF *α*-phase are placed at (100), (020), and (110) planes.

The XRPD spectra changed with the addition of GNPs in PVDF aerogel samples. Notably, the same *α*-phase peaks at (100) and (130) planes disappeared, and the intensity of other *α*-phase peaks sharply reduced and shifted broadly. Moreover, the intensity of (020) peak significantly dropped, and the critical peak at 2*θ* = 20° moved to 2*θ* = 20.7° and displayed wide broad. The *α*-phase transformation into the crystal *β*-phase can be the main reason for the peak shifting at 2*θ* = 20° to 2*θ* = 20.7°, related to (110) and (200) β-phase planes^35^.

Figure 3c) shows the thermal conductivity of neat and GNP-loaded PVDF aerogels as a function of the mid-height sample temperature. At first, it can be noticed that the thermal conductivity increases when GNPs are dispersed inside the polymer matrix, as widely observed in literature^40–42^. In particular, at room temperature the thermal conductivity of neat PVDF samples, G0A and G0B, are 0.121 W/(mK) and 0.262 W/(mK), respectively. These values are close to the ones reported in recent papers^41,42^. With the addition of 7% wt. of GNP, the thermal conductivity at 25 °C rises up to 7.79 W/(mK) for sample G7A and up to 14.23 W/(mK) for sample G7B. This significant enhancement is due to exceptional graphene thermal properties and to the unique 3D structure of GNP loaded composite aerogels, since the interconnected GNP structure acts as an efficient thermal pathway in the polymer matrix^20,41,42^

The measured results of volume reduction due to sample heating after the thermal conductivity tests are displayed in (Fig. 3d). Only neat PVDF aerogel samples and the ones filled with 7% wt. of GNP exhibit a reduction of volume size, due to polymer shrinkage. This effect becomes negligible when a higher amount of GNP is added. In fact, the samples filled with GNPs at 11% and 15% wt. are not affected by any relevant volume reduction. This can be attributed to the fact that for higher GNP concentration, the aerogel is supported mechanically by a skeleton made of a 3D network of GNPs, which prevents the thermally induced shrinkage of the aerogel^39^. On the contrary, for lower GNP concentration, the polymeric nature of the aerogel is predominant over the observed volume reduction.

The mechanical properties of the produced samples were evaluated through in-plane compression tests and the results are reported in (Fig. 3e,f) for A-type and B-type samples, respectively. The compressive curves of fabricated samples can be separated into three regions. A small linear elastic region is observed for strains below 15% for both A-type and B-type aerogel samples. In this region, the pore walls played the elastic deportation roles. The linear elastic region is followed by an extended yield region for applied strains up to 50%. This behavior is associated with the gradual collapse of aerogel pores due to increasing strains^43–46^. The subsequent densification region appear for strains greater than 50%.

**Figure 4:**
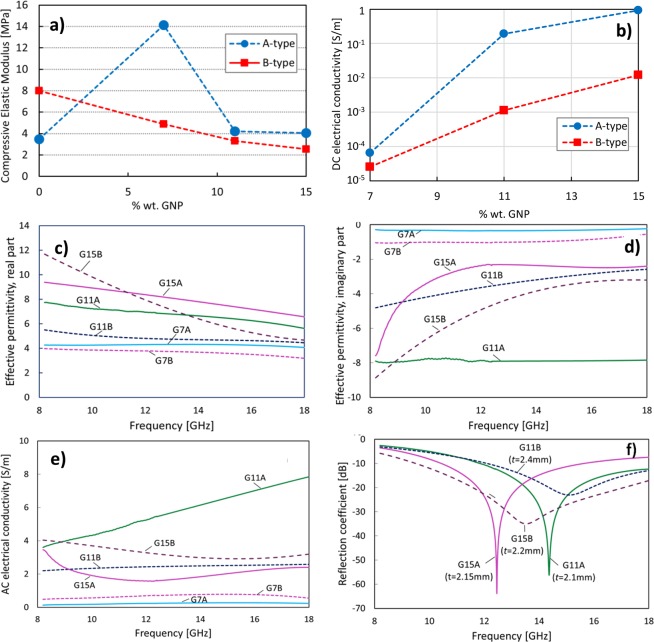
(**a**) Measured compression elastic modulus and (**b**) DC electrical conductivity of the produced samples as a function of the GNP weight content. (**c**) Real part and (**d**) imaginary part of the measured effective complex permittivity, and (**e**) AC electrical conductivity extracted from the imaginary part of the permittivity. (**f**) Reflection coefficient of absorbing panels made of materials G11A, G11B, G15A and G15B backed on a perfect electrically conducting surface.

The compression elastic modulus in the linear elastic region is reported in (Fig. 4a) for the different samples produced at the two different temperatures of 65 °C and 85 °C, as a function of the GNPs % wt. The corresponding measured direct-current (DC) electrical conductivity of the samples is shown in (Fig. 4b). We notice that the process temperature has a relevant effect on both the mechanical and electrical properties of the aerogel. The data reported in (Fig. 4a) reveal that A-Type aerogel produced at lower temperature, for GNP content up to 7% wt. are characterized by a higher compressive elastic modulus than the neat PVDF aerogel. This is reasonably due to the lower porosity of sample G7A and to the larger size of spherulites, as it results from the SEM image of (Fig. 2e). For higher GNP content, we observe a reduction of the compressive elastic modulus, which is representative of the formation of an interconnected GNP framework inside the aerogel, and an enhancement of the DC electrical conductivity. These results are in line with data reported in^19^.

Next, the results of the EM characterizations, carried out after conditioning the samples for 48 h at controlled temperature and humidity, are reported in (Fig. 4c–e). In particular, the frequency spectra of the measured real and imaginary parts of the complex effective permittivity are reported in (Fig. 4c,d), respectively. The measurements have been carried out in the X and Ku frequency bands, i.e. in the 8.2 GHz ÷ 18 GHz frequency range. The alternating-current (AC) electrical conductivity of the sample is reported in (Fig. 4e). From the comparison of data of DC and AC conductivity, it is evident that for GNP concentration up to 7% wt. the produced aerogels behave as lossy dielectrics. Displacement currents are higher in B-type samples processed at 85 °C: this suggest that GNPs are highly integrated in the polymer matrix.

Finally, in (Fig. 4f) EM wave absorption properties of the composite aerogels filled with 11% and 15% wt. of GNPs, are investigated by predicting the reflection coefficient of absorbing panels made of the produced materials backed with perfect electric conducting (PEC) metal plates. The thickness of each absorber has been designed in order to have a minimum reflection peak between 12 and 15 GHz and a maximum bandwidth with reflection coefficient below −20 dB. It is noticed that the best EM wave performance is reached considering sample G15B: with a thickness of 2.2 mm, it is possible to obtain an absorbing panel with a reflection peak at ~13.5 GHz and bandwidth with reflection coefficient lower than −20 dB or −10 dB wider than 6 GHz or 9 GHz, respectively.

## Discussion

A new graphene-based aerogel composite for EM wave absorption, using PVDF as polymer matrix and GNPs as fillers, is presented. An innovative production process has been developed; it does not require chemical modification nor functionalization of the GNPs or of the polymer chains and it is green, since it does not involve the use of acids or bases. All the produced samples have densities and porosities within the range of values for graphene-based polymer composite aerogels. In fact, the maximum measured density, equal to 0.289 g/cm^3^, is well below the densities obtained with MWCNT-filled PVDF aerogels^18^ or with porous PVDF/MWCNTs/graphene composites, ranging between 0.5 and 0.62 g/cm^3^ ^47,19^. The porosity is always greater than 65%, much higher in comparison with PVDF/GNP nanocomposite foams. For instance, in^25^ a porosity of 48.7% is claimed for the PVDF foam filled with 10% wt. of GNPs, while our best produced sample filled with 11% wt. of GNP (i.e. G11B) shows a porosity of 86.8%.

It is worth noting that the porosity increases with the GNP addition, up to a GNP loading of 11% wt., whereas it decreases for higher graphene concentration. In fact, during the phase separation process, the diffusion rate between water and solvent is accelerated by the presence of GNPs^48^, thus originating the porous structure.

However, when the GNP concentration further increases, the GNPs tend to close most of the pores, as observed also in PVDF/GNP nanocomposite films^35^ and confirmed by SEM images of samples filled with 15% wt. of GNPs. This trend is more evident in aerogels produced at lower temperature: (Fig. 2o,p) of sample G15A (process temperature: 65 °C; porosity: 70,8%) show a more compact structure with a higher amount of GNPs closing pores than in (Fig. 2q,r) of sample G15B (process temperature: 85 °C; porosity: 77%). This is probably due to the fact that a higher processing temperature promotes GNP incorporation into the polymer matrix and adhesion of PVDF over the GNP flake’s surfaces.

All the aerogels are characterized by a spherulitic structure, similar to the one observed in GNP-filled PVDF films^35^. Furthermore, the spherulites have an average diameter ranging between 1.5 μm and several micrometers. Similar dimensions were observed in^19^, where MWCNTs and GNPs were added to the PVDF polymer matrix.

The spherulitic morphology of PVDF/GNP composite aerogels as well as of PVDF/GNP films is due to the crystallization of PVDF during liquid-solid phase separation^19^, since GNPs act as nucleating sites for PVDF and facilitate PVDF crystallization, thanks to the special affinity between PVDF molecules and GNP surfaces^35^. This PVDF crystallization results in the phase transformation from *α*-phase to *β*-phase^35^, as confirmed by FTIR and XRD results.

It is also noticed that in B-type samples, the spherulitic size does not change significantly with the considered GNP weight concentrations, whereas in A-type samples the morphology is clearly affected by the GNP content. This is explained with a weaker integration between GNPs and PVDF in samples processed at lower temperature^49^.

Concerning the mechanical properties, we notice that the samples produced at 65 °C exhibit a compressive elastic modulus, as a function of the GNP content, similar to the one reported in^20^: it increases with the GNP per-cent weight fraction up to 7% wt., and then it decreases for higher concentrations. This trend, which is also correlated to the lower porosity and larger spherulite size of sample G7A, as observed in^43,46^, reveals that for a lower GNP content the elastic property of PVDF is predominant over the rigidity of GNPs. Differently, all aerogels produced with a processing temperature of 85 °C are characterized by a decreasing compressive elastic modulus with the GNP content. Actually, in this case, even at 7% wt. of GNPs, the higher rigidity of GNPs prevails over the better elastic properties of the polymer, since the 3D-GNP network is fully integrated in to PVDF, due to the enhanced adhesion of PVDF over the GNP flake surface, promoted by the higher processing temperature.

The GNP-loaded aerogels show a significant enhancement of both thermal and DC electrical conductivities with respect to neat PVDF aerogel samples. This enhancement is due to GNP excellent thermal and electrical properties and to the unique 3D structure of GNP loaded composite aerogels, since the interconnected GNP network acts as an efficient thermal and electrical pathway through the polymer matrix^20,41,42^. Furthermore, it is worth to notice that A-type PVDF-GNP samples are characterized by higher thermal conductivities in comparison to B-type PVDF-GNP samples. This confirms the hypothesis that aerogels processed at lower temperature maintain the double nature of the two phases (i.e. PVDF and GNPs), whereas the ones processed at 85 °C are characterized by a better incorporation of GNPs in PVDF and, consequently, are characterized by a lower thermal conductivity and a lower DC electrical conductivity than the samples processed at 65 °C. The maximum electrical conductivity of 1 S/m is measured for sample G15A and is reasonably due to the interconnected GNP structure characterizing this sample.

The EM properties are strongly affected by GNPs’ addition. Samples G7A and G7B, with a lower GNP content, are characterized by an effective complex permittivity which has a dominant real part, meaning that the dielectric behavior is predominant. As the GNP content increases, the amount of GNP connecting directly the micro-sized spherulites increases. Thus, due to their bidimensional shape, GNPs act at the same time as micro- and nano-fillers, affecting both real and imaginary part of the effective complex permittivity^9^. Samples filled with 11% and 15% wt. of GNPs are characterized by an AC electrical conductivity of a few siemens-per-meter and, at the same time, by a ratio between the imaginary and real parts of the effective permittivity greater than 0.6 and approaching 1 for sample G15B.

Finally, looking at the EM wave absorption properties, we noticed that the best performance in terms of bandwidth with minimum reflection coefficient below −20 dB was obtained with sample G15B. In fact, sample G15B is characterized by a nearly constant AC electrical conductivity and by a real part of the effective permittivity decreasing with frequency, thus enabling the wide-band absorption properties in the 8 ÷ 18 GHz range^9^.

Such EM absorption performance is comparable with the one obtained with graphene foam^28^ and is particularly relevant with respect to the one obtained with graphene-silicon carbide aerogels^29^ or with other porous polymer composites, such as helical conducting polypyrrole nanostructures^30^, hierarchically porous cobalt based zeolitic imidazolate frameworks^31^ or coaxial silver-polypyrrole porous nanocomposites^32^. We also point out that sample G15B is water repellent, therefore it is particularly suitable for the realization of EM absorbers with a stable response, since its EM properties are not affected by humidity.

## Method

### Sample preparation

To produce GNPs, first, a graphite intercalation compound (GIC) underwent a thermal shock at 1150 °C, where the obtained material is known as thermally expanded graphite oxide (TEGO)^9^. Neat PVDF aerogel samples were fabricated by the solution method. Meanwhile, PVDF was dissolved in N,N dimethylformamide (DMF) for two hours by magnetic stirring at 65 °C (A-type samples) or 85 °C (B-type samples). The so obtained PVDF-DMF solution was poured into glass beakers and left at room temperature to expedite polymer gelification for 12 h. Over this time frame, polymer chains rearranged themselves into a physical 3D network. Then, a solution of deionized water and methanol was poured onto samples surface to allow DMF removal. Finally, after 48 fours, samples were freeze-dried at −40 °C over a two days period.

In order to produce graphene-based aerogel nanocomposites, TEGOs were mixed with DMF, and the mixed suspension was tip sonicated for 20 minutes using an ultrasonic processor. This step increases the GNP dispersion and reduces the amount of agglomerates^14^. A homogeneous suspension of GNPs in DMF was thus obtained and PVDF, in powder form, was added to the suspension of GNPs in DMF. Then PVDF was dissolved in the resulting mixture through two hours-magnetic stirring at 65 °C (A-type samples) or 85 °C (B-type samples). The temperature is crucial in the PVDF aerogel production process. In particular, the temperature of 65 °C has been chosen as an optimum temperature for obtaining a homogenous structure of the PVDF polymer. On the other hand, adhesion between GNPs and PVDF matrix is enhanced when setting 85 °C as the process temperature^35^.

Finally, the obtained PVDF-DMF-GNP mixtures were subjected to gelification, DMF removal, and freeze-drying following the same above-mentioned steps for neat PVDF aerogel. Furthermore, graphene nanoplatelets favored polymer chains physical crosslinking during the gelification time, since graphene can act as a bridge between polymer chains.

### Morphological characterization

Morphology was assessed through Field Emission Scanning Electron Microscopy (FE-SEM) using a Zeiss Auriga platform, available at Sapienza SNN-Lab. Samples were scanned at 5 keV accelerating voltage, and images were obtained using secondary electrons detectors. In order to prevent electric charging, specimens were sputter coated with a 20 nm Cr layer before morphological analysis.

### Fourier transform infrared spectrometer

The FTIR spectra were performed at room temperature by using an FTIR spectrometer (Vertex 70 v, Bruker Optics GmbH). The spectra were recorded in the 4000–400 cm^−1^ wavenumber range, for crystalline phases’ identification.

### X-ray powder diffraction

XRPD data were recorded on a Bruker AXS D8 Advance X-Ray diffractometer, employing the 1.54 Å Cu Kα rediation, operating in θ/θ transmission mode. The instrument is fitted with incident-beam focusing Göbel mirrors, a capillary stage, and a position sensitive detector VÅntec-1. Samples were loaded and gently packed inside borosilicate-glass capillaries with 1 mm diameter. Patterns were measured in the 7–60° 2θ angular range, 0.022° 2θ step size, and 4 s counting time.

### Mechanical characterization

Compression tests were conducted using a dual column table top electromechanical universal testing machine (Model Instron 3366), equipped with a ± 10 N load cell for static applications (Model 2530-428), directly mounted to the moving frame’s crosshead, and with two different stainless steel hardened/chrome plated plane platens.

Thickness and lateral dimensions of the produced samples were firstly measured with a digital caliper (Mitutoyo). Then, the specimens are placed on the centre of the lower 150 mm diameter platen (Model 2501-163) and compressed progressively at a constant crosshead speed of 1 mm/min by the upper 57 mm diameter platen (Model 2501-108), which is attached to the load cell.

The stress (kPa) vs strain (mm/mm) diagram of the material samples were obtained up to a maximum compression of 0.75 mm/mm: the uniformly applied force measured by the load cell is used to calculate the stress as the ratio of the applied load to the initial specimen cross-sectional area; the strain is obtained dividing the change in thickness (deformation) of the sample by its initial thickness.

### Thermal characterization

The test method is aimed at measuring the thermal conductivity through the determination of the heat flux, following ASTM 1530-11. The method used is the quasi-steady state test method^50^ through the measurement of three temperatures. The samples have been guarded and insulated in order to avoid thermal losses. Then, the specific heat has been computed at different temperatures.

### Electrical characterization

The opposite faces of the rectangular-shaped samples are contacted using a thin silver-paint layer (Electrolube) and a conductive adhesive (CircuitWorks) to bond tin-coated copper wires. Then, the samples are cured in a desiccator at room temperature for two days. The electrical resistance of the film is measured using a Keithley 6221 dc/ac current source connected to a Keithley 2182a nano-voltmeter. Then, the DC electrical conductivity is extracted.

### Electromagnetic characterization

The composite aerogel samples were inserted in X-band and Ku-band rectangular aluminum flanges and accurately finished. Then, all samples were dried for 24 h at controlled temperature and humidity. The scattering parameters of the filled flanges were measured with a vector network analyzer (Anritsu Vector Star MS4647 A) using two different sets of waveguides, in X- and Ku-band, respectively, in order to cover the 8 ÷ 18 GHz frequency range. The complex effective permittivity $\bar{\varepsilon }=\varepsilon ^{\prime} +j\varepsilon ^{\prime\prime}$ of the composites is finally extracted from the measured parameters, according to^51^. Being *ε*′ the real part and *ε*″ the imaginary part of $\bar{\varepsilon }$, the AC effective electrical conductivity *σ* is derived from *ε*″ as:

1 $$\sigma =\omega {\varepsilon }_{0}|\varepsilon ^{\prime\prime} |$$

and the dielectric loss tangent tan*δ* of the produced samples is computed as:

2 $$\tan \,\delta =|\varepsilon ^{\prime\prime} |/\varepsilon ^{\prime} $$

### Reflection coefficient prediction

We consider radar absorbing panels made of the produced materials backed with PEC plates. The reflection coefficient *R* of such panels illuminated by a plane wave with normal incidence can be expressed as:

3 $$R=20\,\log |\frac{1-{\eta }_{0}{Y}_{in}}{1+{\eta }_{0}{Y}_{in}}|$$

where *η*_0_ = 377 Ω is the free space wave impedance and *Y*_*in*_ is the input admittance, given by:

5 $${Y}_{in}=\frac{{\Phi }_{22}}{{\Phi }_{12}}$$

in which Φ_22_ and Φ_12_ are the coefficients of the material output-to-input transmission matrix [Φ], and are computed applying the well-known transmission line model as described in^9^, as function of the effective complex permittivity of the sample and of the thickness of the material. In particular, the thickness of each absorber has been optimized in order to have a minimum reflection peak between 12 and 15 GHz and a maximum bandwidth with reflection coefficient below −20 dB.

## Supplementary information

Supplementary Information
